# Interleukin-33 Promotes Cell Survival via p38 MAPK-Mediated Interleukin-6 Gene Expression and Release in Pediatric AML

**DOI:** 10.3389/fimmu.2020.595053

**Published:** 2020-11-26

**Authors:** Yiqian Wang, Haibo Su, Muxia Yan, Li Zhang, Jiancheng Tang, Quanxin Li, Xiaoqiong Gu, Qing Gong

**Affiliations:** ^1^ Department of Biochemistry and Molecular Biology, GMU-GIBH Joint School of Life Sciences, Guangzhou Medical University, Guangzhou, China; ^2^ Department of Hematology, Guangzhou Women and Children’s Medical Center, Guangzhou Medical University, Guangzhou, China; ^3^ Department of Anesthesiology, Second Affiliated Hospital of Guangzhou Medical University, Guangzhou, China; ^4^ Department of Blood Transfusion, Clinical Biological Resource Bank and Clinical Lab, Guangzhou Institute of Pediatrics, Guangzhou Women and Children’s Medical Center, Guangzhou Medical University, Guangzhou, China

**Keywords:** acute myeloid leukemia (AML), interleukin 1 receptor Like 1 (IL1RL1), interleukin-33 (IL-33), p38 mitogen-activated protein kinase (MAPK), interleukin-6 (IL-6)

## Abstract

Acute myeloid leukemia (AML) is a fatal disease characterized by the accumulation of immature myeloid blasts in the bone marrow (BM). Cytokine provide signals for leukemia cells to improve their survival in the BM microenvironment. Previously, we identified interleukin-33 (IL-33) as a promoter of cell survival in a human AML cell line and primary mouse leukemia cells. In this study, we report that the cell surface expression of IL-33–specific receptor, Interleukin 1 Receptor Like 1 (IL1RL1), is elevated in BM cells from AML patients at diagnosis, and the serum level of IL-33 in AML patients is higher than that of healthy donor controls. Moreover, IL-33 levels are found to be positively associated with IL-6 levels in pediatric patients with AML. *In vitro*, IL-33 treatment increased IL-6 mRNA expression and protein level in BM and peripheral blood (PB) cells from AML patients. Evidence was also provided that IL-33 inhibits cell apoptosis by activating p38 mitogen-activated protein kinase (MAPK) pathway using human AML cell line and AML patient samples. Finally, we confirmed that IL-33 activated IL-6 expression in a manner that required p38 MAPK pathway using clinical AML samples. Taken together, we identified a potential mechanism of IL-33–mediated survival involving p38 MAPK in pediatric AML patients that would facilitate future drug development.

## Introduction

Acute myeloid leukemia (AML) is a heterogeneous disease characterized by specific chromosomal abnormalities, and it accounts for approximately 20% of pediatric leukemias. In the past few decades, there has been incremental progress towards curing pediatric AML through refined treatment regimens and improved supportive care (1). Although event-free survival (EFS) rates for children with newly diagnosed AML ranges from 50% to 60%, currently, there is no standard treatment for relapsed or refractory AML (2, 3). Thus, achieving better outcomes remains an important issue for the therapeutic success of treating pediatric AML patients.

The tumor microenvironment can affect the growth, survival and drug resistance of cancer cells (4, 5). Cytokines existing in tumor microenvironment are known to have profound effects in promoting cancer progression (6). Dysregulation of the complicated cytokine network creates a pro-tumorigenic microenvironment. In AML, the most relevant microenvironment is within the bone marrow (BM) (7). A critical role of the BM and the development of AML has been reported in various studies (8–11). However, the relationship between cytokines in the BM and leukemia cell activity are not fully understood. Interleukin 33 (IL-33) belongs to IL-1 family of cytokines, and participates in various biological activities, including inflammation, immune response and the infection process (12). Recent studies have identified IL-33 as an important regulator during the development of hematologic malignancies. In chronic myeloid leukemia (CML) cells expressing BCR-ABL1, IL-33 increases the proliferation and helps leukemia cells exert resistance to the inhibitor imatinib (13). Of note, other reports suggest that IL-33 has an immunosuppressive effect by regulating immune cell activities in AML (14, 15).

Previously, we found that IL1RL1 is highly expressed in leukemia cells expressing *CBFB-MYH11* (16, 17). Further, we demonstrated that exogenous IL-33 treatment inhibited apoptosis of primary mouse leukemia cells as well as the human AML cell line, HL-60 (18). Further investigation revealed that the IL-33/IL1RL1 axis exerts the anti-apoptotic effect by potentially activating p38 MAPK pathway. In addition, we found that the addition of exogenous IL-33 promotes the mRNA expression of the inflammatory cytokine, IL-6, in mouse leukemia cells by *in vitro* analysis (18). However, the role of IL-33/IL1RL1 axis in primary human AML samples has not been reported so far.

In the present study, we show that both IL-33 and IL1RL1 are upregulated in AML patient samples as compared healthy donors. By treating primary AML samples with IL-33 and anti-IL-33 antibody, we found that IL-33 led to IL-6 expression and production. Consistent to our previous study using AML cell line, we found that IL-33 inhibited apoptosis via activating the p38 MAPK pathway by in human AML patient samples. Finally, we found that the inhibition of p38 MAPK signaling abrogated the upregulation of IL-6 expression and secretion mediated by IL-33 in BM cells from AML patients. Collectively, our data demonstrate that IL-33 promotes AML cell survival and stimulates cytokine production via the p38 MAPK pathway in pediatric patients with AML.

## Materials and Methods

### Patient Samples

Freshly collected or frozen bone marrow (BM) or peripheral blood (PB) samples were obtained from AML patients or healthy donors in accordance with the Declaration of Helsinki, and was approved by the Guangzhou Women and Children Medical Center Ethics Committee (ethics number: 2020-39500). All patients were risk-classified according to the latest World Health Organization (WHO) classification (19, 20). The medical history of AML patients and healthy control subjects was obtained, and all patients provided written informed consent. Mononuclear cells (MNCs) from PB and BM samples were obtained using Human Mononuclear Cells Separation Medium 1.077 (Dongfang Huahui) and used for subsequent experiments. Normal samples correspond to steady-state serum and BM samples from healthy donors were collected after informed consent.

### Tissue Culture

For *in vitro* studies, both IL-33 (Sino Biological) and anti-IL-33 antibody (Biovision) were used at 100 ng/ml, and SB203580 used at 20 μM. The human AML cell line HL-60, BMMCs, and PBMCs were grown in RPMI1640 (ATCC) supplemented with 20% FBS, 2mM L-Glutamine, and 1% penicillin-streptomycin. All cells were grown at 37 °C and 5% CO2.

### Enzyme-Linked Immunosorbent Assay and CBA Assay

Enzyme-linked immunosorbent assay (ELISA) (4A Biotech) was performed on cell culture supernatant and serum in AML patients and the healthy donors according to the manufacturer’s protocol. Briefly, the samples and the standard samples were incubated with horseradish peroxidase (HRP)-conjugated antibodies. The substrate TMB was added which became blue under the catalytic action of peroxidase. Absorbance was measured at 450 nm. The specific concentrations were calculated by using the software program CurveExpert version 1.4.

The *in vitro* IL-6 levels were evaluated in the collected supernatants using a cytometric bead array (CBA) for human IL-6 cytokine (BD Biosciences). The data were acquired using a CytoFlex S cytometer (Beckman Coulter) and analyzed by CytExpert 2.3 software (Beckman Coulter).

### Quantitative RT-PCR Analysis

Total RNA was isolated from cells using AG RNAex Pro Reagent (ACCURATE BIOTECHNOLOGY) according to the manufacturer’s specifications, synthesized into cDNA using reverse transcription PCR (ACCURATE BIOTECHNOLOGY). Real-time quantitative analysis (qRT-PCR) was performed using SYBR Green Premix Pro Taq HS qPCR Kit (ACCURATE BIOTECHNOLOGY) with specific primers for *Actb*, and *IL-6* (ACCURATE BIOTECHNOLOGY). The real-time PCR was run on a LightCycler® 96 Instrument (Roche). Expression levels of genes were normalized to *Actb* mRNA. The 2–ΔΔCT method was used to compare the relative expression levels of mRNA among different groups. Primers used for quantitative PCR include *IL-6f*: 5′-TGAACTCCTTCTCCACAAGCG-3′ and IL-6r: 5′-TGGAATCTTCTCCTGGGGGTA-3′. *Actbf*:5′-GGATGCAGAAGGAGATCACTG-3′ and *Actbr*:5′-CGATCCACACGGAGTACTTG-3′) (21, 22).

### Western Blot Analysis

Total proteins were extracted with the SDS lysis buffer (Beyotime) or RIPA buffer (KeyGEN). Samples were subjected to electrophoresis in SDS-PAGE gels and then transferred to a PVDF membrane (Millipore) for antibody blotting. The membrane was incubated with the primary antibody overnight, followed by 1-h incubation with horseradish peroxidase (HRP)-conjugated anti-rabbit or anti-mouse secondary antibodies at room temperature, washed and incubated with chemiluminescence (ECL) reagent (Beyotime). The following primary antibodies were used for Western blotting: anti-p-p38 MAPK (Immunoway), anti-p38 (Immunoway), anti-pATF2 (Immunoway) and GAPDH (Abways). Protein bands were quantified by densitometric scanning and normalized to the GAPDH loading control. For full scans of Western blots see **Supplemental Figures S2–S4**.

### FACS Analysis

Primary samples were stained with APC conjugated anti-IL1RL1 (BD Biosciences). For the measurement of apoptosis, we used the FITC-conjugated Annexin V (Procell). Cells were analyzed using CytoFlex S (Beckman Coulter) within 1 h. Cell cycle was determined by propidium iodide (PI) staining after cells were fixed with ice-cold 70% ethanol for at least 30 min at 4°C. FACS data was analyzed by CytExpert 2.3 software (Beckman Coulter) and ModFit software (Verity Software).

### Statistical Analysis

P-values and Pearson’s correlation coefficients were calculated by Prism 8.0.1 (GraphPad software Inc.). The statistical significance of differences in mean values was analyzed by paired *t* test. One-way ANOVA with Tukey post hoc test was used to perform multiple comparisons between experimental conditions. Data are represented as mean values ± standard deviation. *P* values < 0.05 were considered statistically significant.

## Results

### Interleukin 1 Receptor Like 1 Expression and Serum Interleukin-33 Level Are Higher in Patients With Acute Myeloid Leukemia

**Figure 1:**
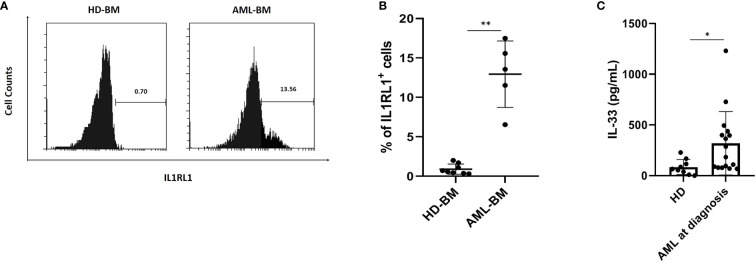
AML patients at diagnosis have elevated IL1RL1 expression and IL-33 level in the serum. **(A, B)** IL1RL1 expression is increased on the cell surface of BM cells from AML cohorts. BMMCs from patients with AML and healthy donors were analyzed for IL1RL1 expression. Each symbol represents one healthy donor or patient with AML. **(C)** ELISA assay was used to measure IL-33 levels in serum from AML patients at diagnosis and normal children. Each symbol represents one healthy donor or patient with AML. n*≥*3; *P < 0.05; **P < 0.01. Paired *t* test.

To address the question whether IL1RL1 cell surface expression is upregulated in primary AML samples, we isolated MNCs from the BM of AML patients (AML cohort; n = 5) as well as healthy donors (HD cohort; n = 8), which were then stained with anti-IL1RL1. Flow-cytometric analysis revealed substantial IL1RL1 expression from most AML patients, while samples from healthy donors were expressing this receptor at very low levels (**Figures 1A, B** and **Supplemental Figure S1**). By further analyzing the IL-33 profile in the serum collected from AML patients (AML cohort; n = 16) at diagnosis and from healthy donors (HD; n = 9). We found that IL-33 levels were significantly elevated in AML patients compared with normal controls (**Figure 1C**). Importantly, in the patient group, the concentrations of IL-33 were elevated in a subset of AML patient samples, indicating that the variability may provide the ability to evaluate for potential relationships between IL-33 levels and clinical outcomes. Taken together, these results suggest that increased serum level of IL-33 along with the overexpression of IL1RL1 receptor in the BM could be a plausible mechanism for AML maintenance.

### Baseline Concentration of Interleukin-6 Is Positively Correlated With Interleukin-33 in the Serum and Interleukin-33 Induces Interleukin-6 Expression in Primary Acute Myeloid Leukemia Cells

**Figure 2:**
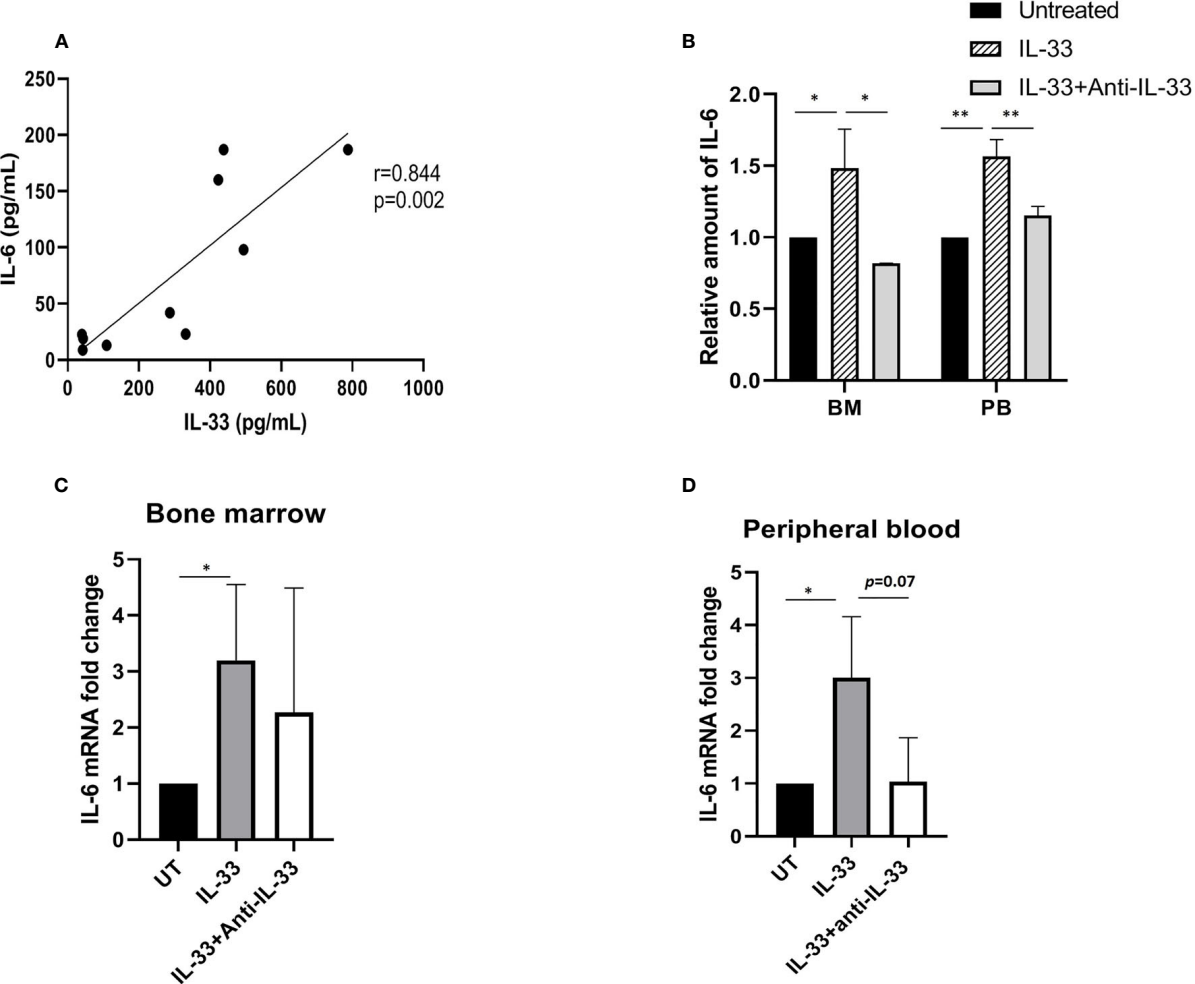
IL-33 induces IL-6 expression and secretion in primary AML cells. **(A)** The relationship between IL-33 and IL-6. Spearman rank correlation analysis was performed to evaluate the correlation of serum IL-33 with IL-6 in patients with AML at diagnosis (n = 10). **(B)** PBMCs and BMMCs from patients diagnosed with AML were incubated for 72 h with IL-33 (100 ng/ml) or combined with anti-IL-33 (100 ng/ml). CBA was used to simultaneously measure IL-6 in supernatants from cell cultures, and plotted as fold change compared with untreated samples. **(C, D)** RNA/cDNA expression of cells from **(B)** was analyzed using quantitative real-time PCR using *Actb* as a reference control. Data are plotted as relative gene expression compared with the untreated control. A significant difference in the mean IL-6 mRNA expression between compared with untreated cells was found in both BM and PB samples. n≥3; *P < 0.05; **P < 0.01. One-way ANOVA test.

Previously, we demonstrated that exogenous IL-33 increases the expression of IL-6 using primary mouse leukemia cells expressing *Cbfb-MYH11*, which indicates that IL-33 may induce cytokine release to inhibit AML cell apoptosis (18). To further elucidate the cytokine milieu associated with IL-33 in AML patients, we firstly measured the levels of both IL-33 and IL-6 from AML patients at diagnosis. We found that the serum concentrations of IL-6 showed a significantly positive correlation with IL-33 levels at baseline (r = 0.844, *p* = 0.002) (**Figure 2A**). Next, to examine whether IL-33 could provoke enhanced IL-6 expression in primary AML samples, we treated BM and PB cells with IL-33 or in combination with anti-IL-33 antibody for 72 h in culture and determined IL-6 expression. We found that IL-6 secretion was elevated in response to IL-33, while antibody blockade of IL-33 abrogated the effect on increased IL-6 secretion mediated by IL-33 in both BM and PB (**Figure 2B**). Additionally, IL-33 treatment led to significantly increased IL-6 mRNA expression as compared to untreated cells. Interestingly, cells treated with the combination of IL-33 with anti-IL-33 antibody showed a trend towards decreased IL-6 mRNA expression as compared to IL-33 treatment alone, although this difference was not statistically significant in either BM or PB (**Figures 2C, D**). Together, our data suggest that IL-33 might work with IL-6 in the local and systemic circulation to support the maintenance of leukemia cells. Importantly, the IL-33/IL1RL1 axis also stimulates cytokine production in patients with AML.

### Interleukin-33 Activates p38 Mitogen-Activated Protein Kinase Pathway in Primary Acute Myeloid Leukemia Samples

**Figure 3:**
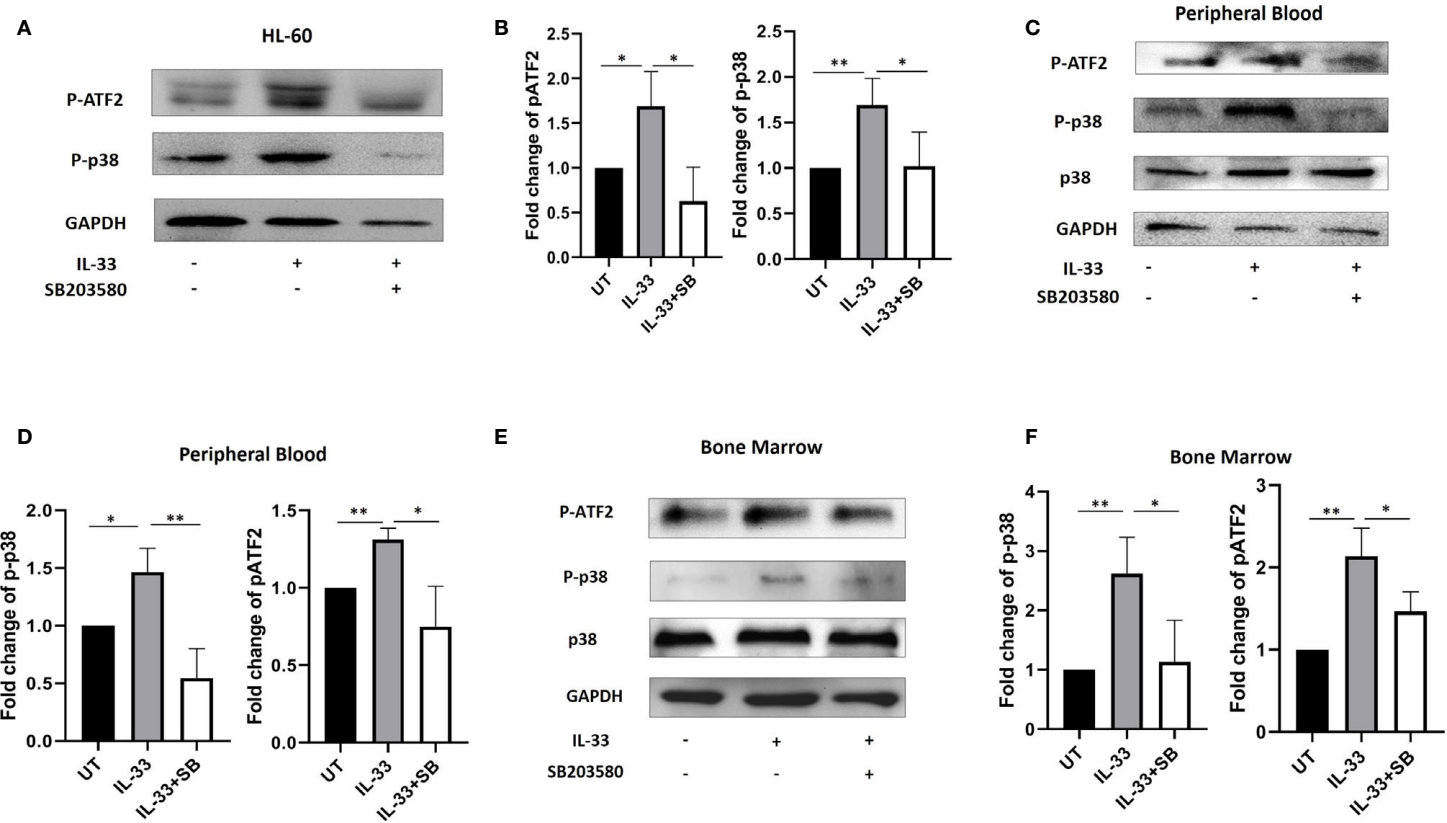
IL-33 activates p38 MAPK pathway in primary AML samples. **(A, B)** The AML cell line HL-60 were treated with IL-33 (100 ng/ml) or in combination with SB (20 µM). P-p38, pATF2, or GAPDH protein expression was probed by Western blot analysis. The bar graph shows the quantification of p-p38 and pATF2 protein in all groups. **(C–F)** PBMCs and BMMCs from patients diagnosed with AML were incubated for 72 h with IL-33 (100 ng/ml) or in combination with SB (20 µM). P-p38, p38, pATF2, or GAPDH protein expression was probed by Western blot analysis. The bar graphs show the quantification of p-p38 and pATF2 protein in all groups. n≥3*;* *P < 0.05; **P < 0.05. One-way ANOVA test.

IL-33 is able to induce activation of the p38 MAPK pathway in both human natural killer (NK) cells and macrophages (23, 24). In addition, our previous work demonstrated that IL-33 increases the phosphorylation of p38 MAPK, which was inhibited by p38 MAPK inhibitor, SB203580 (SB) in the human AML cell line HL-60 (18). To further investigate this, we treated HL-60 cells with IL-33 alone or in combination with SB in culture. We then measured the phosphorylation levels of p38 MAPK and activating transcription factor (ATF2, Thr69/71), one of the downstream molecules in the MAPK signal pathway. We found that IL-33 significantly increased the phosphorylation of both p38 MAPK and ATF2 as compared to the untreated cells, while SB significantly reduced IL-33-induced p38 MAPK and ATF2 phosphorylation (**Figures 3A, B**). To determine if IL-33 activates p38 MAPK pathway in AML patient samples, primary AML cells from BM and PB were cultured and treated with IL-33 alone or in combination with SB for 72 h. Phosphorylation of p38 MAPK and ATF2 were measured by Western blot. We found that IL-33 treatment stimulated the phosphorylation of both p38 MAPK and ATF2, whereas the addition of SB diminished the effect of IL-33-induced activation of p38 and ATF2 (**Figures 3C–F**). These results indicate that IL-33 can enhance the activation of p38 MAPK pathway in primary AML cells from both BM and PB. The data imply that IL-33-mediated p38 MAPK activation may be required by AML cells for the maintenance.

### Interleukin-33 Promotes Cell Survival via p38 Mitogen-Activated Protein Kinase Pathway in Primary Acute Myeloid Leukemia Samples

**Figure 4:**
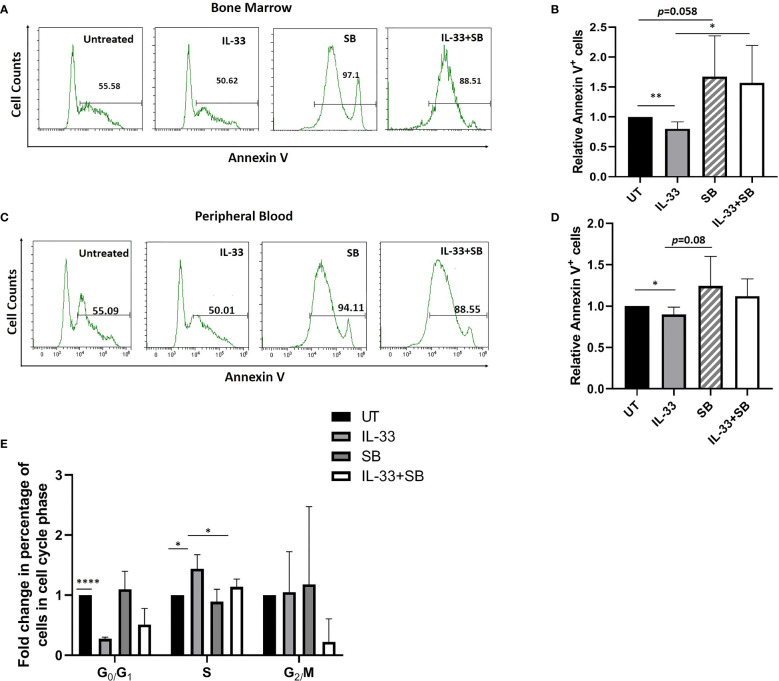
IL-33 promotes cell survival via p38 MAPK pathway in primary AML samples. BMMCs **(A, B)** and PBMCs **(C, D)** from pediatric patients with AML were cultured with IL-33 (100 ng/ml), or SB (20 µM) alone, or in combination for 72 h, and apoptosis was measured by Annexin V staining. Bar graphs show the relative Annexin V staining of leukemia cells as compared to the untreated cells. **(E)** BMMCs from AML patients were analyzed for cell cycle status. Bar graph shows the relative percentage of leukemia cells in the indicated phase of the cell cycle after culture for 72 h in the presence of IL-33 (100 ng/ml), SB (20 µM) or in combination, compared to the untreated cells. n≥3*;* *P < 0.05; **P < 0.05; ****P < .0001. One-way ANOVA test.

To test whether IL-33 influences cell survival via p38 signaling in primary samples, we treated BM and PB cells from AML patients with IL-33 or SB alone, or in combination in culture for 72 h and measured apoptosis and cell cycle status. We found that treatment with IL-33 led to a significant decrease in apoptosis, as compared to the untreated cells in both BM and PB, while SB blocked the anti-apoptotic effect mediated by IL-33 in BM. The combination of IL-33 and SB caused a trend towards higher apoptotic level as compared to IL-33 treatment alone in PB, although this was not statistically significant (**Figures 4A–D**).

By performing cell cycle analysis using BM samples, we found that IL-33 alone caused a significant increase in cells in S phase as well as a significant decrease in G0/G1 phase, as compared to the untreated control, implying that IL-33 can induce proliferation. Moreover, the combination of IL-33 and SB decreased the proportion of cells in S phase as observed with IL-33 alone. Importantly, we found that there was a statistically significant decrease in cells in G0/G1 phase in IL-33-treated cells, as compared to the untreated cells (**Figure 4E**). These results are consistent with our earlier work showing that IL-33 inhibits apoptosis via activating p38 MAPK and upregulates the percentage of cells in S phase in primary mouse leukemia cells and in human AML cell lines (16, 18). Together, our data indicate that IL-33 promotes cell survival by stimulating p38 MAPK pathway in AML patient samples.

### P38 Mitogen-Activated Protein Kinase Contributes to IL-33-Induced Interleukin-6 Expression in Primary Human Samples

**Figure 5:**
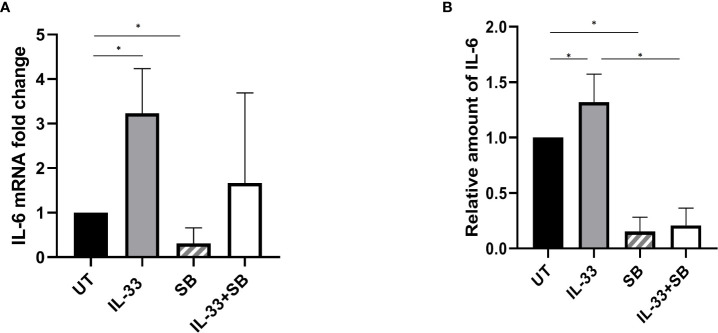
p38 MAPK pathway participates in IL-33-stimulated IL-6 expression and release. BMMCs from pediatric patients with AML were cultured with IL-33 (100 ng/ml), or SB (20 µM) alone, or in combination for 72 h. **(A)** RNA/cDNA expression of cells was analyzed using quantitative real-time PCR using *Actb* as a reference control. Data are plotted as relative gene expression compared with the untreated control. **(B)** CBA was used to simultaneously measure IL-6 in supernatants from cell cultures, and plotted as fold change compared with untreated samples. n≥3; *P < 0.05. One-way ANOVA test.

Having demonstrated that IL-33 has a pro-survival role in AML samples by activating p38 and provoking IL-6 expression, we hypothesized that IL-33 may activate IL-6 expression in a manner that requires p38 MAPK activation. To test this, we treated BM-MNCs from AML patients with IL-33, SB alone or in combination in culture for 72 h and isolated RNA for qRT-PCR analysis. We observed that there was significantly increased expression of IL-6 in IL-33-treated cells while SB cased a significant decrease in IL-6 mRNA expression, as compared to the untreated (**Figure 5A**). Cells with the combination of IL-33 and SB showed a trend towards decreased IL-6 mRNA expression compared to those with IL-33 treatment alone, although this difference was not statistically significant. This result indicates that IL-33 enhances cytokine expression in primary human AML cells. We next sought to examine whether IL-33 could enhance IL-6 release by leukemia cells using the same patient group. We measured the levels of IL-6 in the media with a CBA-based assay after treating BM samples as described above for 72 h. We observed that IL-33 significantly increased IL-6 release as compared to the untreated cells, whereas SB treatment diminished the enhancing effect of IL-33 on IL-6 secretion (**Figure 5B**). These results led us to conclude that p38 MAPK pathway is necessary for IL-33 to induce IL-6 expression and release by leukemia cell from primary AML BM samples.

## Discussion

Over the past decades, there has been a significant improvement in treating pediatric AML (25). However, the outcomes of pediatric AML still remains unsatisfactory, and around 30% of patients relapse (26). Therefore, novel therapeutic approaches to treat childhood AML are required. In recent years, a critical role of cytokines during AML development has been described (27, 28). IL-33 is a recently identified cytokine that belongs to the IL-1 cytokine family (12). Previously, we showed that IL1RL1, the receptor of IL-33, is highly expressed in leukemia cells expressing *Cbfb-MYH11*, and exogenous treatment with IL-33 promoted the survival of both primary mouse leukemia cells and human AML cell lines *in vitro* (16, 18). Further investigation revealed that the IL-33/IL1RL1 axis can inhibit leukemia cell apoptosis via phosphorylation of p38 MAPK (18). However, so far, no clinical studies have demonstrated the role of IL-33/IL1RL1 axis in primary pediatric AML cells. Thus, in this study we further examined the expression of IL1RL1 and IL-33 levels using clinical data, and the mechanism of IL-33-mediated p38 MAPK stimulation in the maintenance of AML pediatric patient cells.

By performing FACS, we first showed that the IL-33 receptor IL1RL1 was constitutively expressed in BM cells from pediatric AML patients at diagnosis, whereas BM cells from healthy controls do not appear to express IL1RL1. In accordance with this observation, we found that there were elevated levels of IL-33 in the serum from AML samples, as compared with HD cohorts. These results imply that IL-33 may play an important role in maintaining AML growth. Since accurate risk stratification is critical for determining the intensity of therapy, our data generate the question of whether there is a relationship between IL-33 and prognosis in AML patients. Thus, precisely tracking individual AML patients would provide additional information and better delineate the correlation between IL-33 levels and clinical outcomes.

Our previous work suggests that IL-33 leads to a decrease in apoptosis, potentially through stimulation of p38 MAPK pathway in the human AML cell line HL-60 (18). In cardiomyocyte, cerebral cells and eosinophils, IL-33 plays a central role in suppressing apoptosis or promoting survival (29–32). Additionally, several studies have demonstrated that IL-33 induces type-1 and -2 cytokine expression via p38 MAPK signaling in innate lymphoid cells or natural killer cells (33, 34). Furthermore, IL-33 was shown to trigger both the mRNA and the protein levels of IL-6, a pro-inflammatory cytokine in the context of different diseases (35–38). Thus, one possible mechanism by which IL-33 inhibits apoptosis in AML could be by inducing IL-6 production through activating p38 MAPK pathway. To this point, we showed that IL-33 increased the phosphorylation of both p38 and ATF2 in primary AML samples, which correlated with increased cell survival. Importantly, the addition of a p38 MAPK inhibitor, SB203580, significantly inhibited the phosphorylation of p38 and ATF2, while simultaneously abrogated the IL-33-induced decrease in apoptosis. Of note,SB203580 reduced the expression and release of IL-6 stimulated by IL-33. Therefore, our data indicate that p38 MAPK pathway is involved in an IL-33-mediated anti-apoptotic pathway in AML patient samples, potentially by stimulating IL-6 expression and secretion. These works also imply that other p38 MAPK-stimulated mediators, including ATF2 may also be important for the pro-survival effect of IL-33 in AML. In our study, we observed that SB203580 in combination with IL-33 reduced p38 MAPK phosphorylation as compared with IL-33 treatment alone. In fact, SB203580 has been known to prevent p38 MAPK activity by inhibiting p38 MAPK catalytic activity but not affecting the phosphorylation of p38 MAPK (39). However, a series of studies have suggested autophosphorylation as an alternative p38 MAPK activation (40, 41). The observed discrepancy between our analysis and previous report indicates that IL-33 may be able to induce p38 MAPK autophosphorylation. Thus, future investigation is warranted.

ATF2 is a well-known stimulator of activator protein 1 (AP-1) transcription factors, both of which are involved in regulating cell cycle, proliferation and pathogenesis in the context of multiple diseases (42, 43). By interacting with ATF2, one of AP-1 complex, c-Jun, has been found to play an important role during apoptosis progression in lymphocytic leukemia cells and participates in drug responses in AML cells (43, 44). Thus, it is conceivable that IL-33 selects p38-dependent downstream effectors to inhibit cell apoptosis, and stimulating the release of cytokine. Future direction should focus on investigating how the p38 MAPK pathway regulates transcription factors including ATF2 and AP-1 complex, which could help in future drug development.

**Figure 6:**
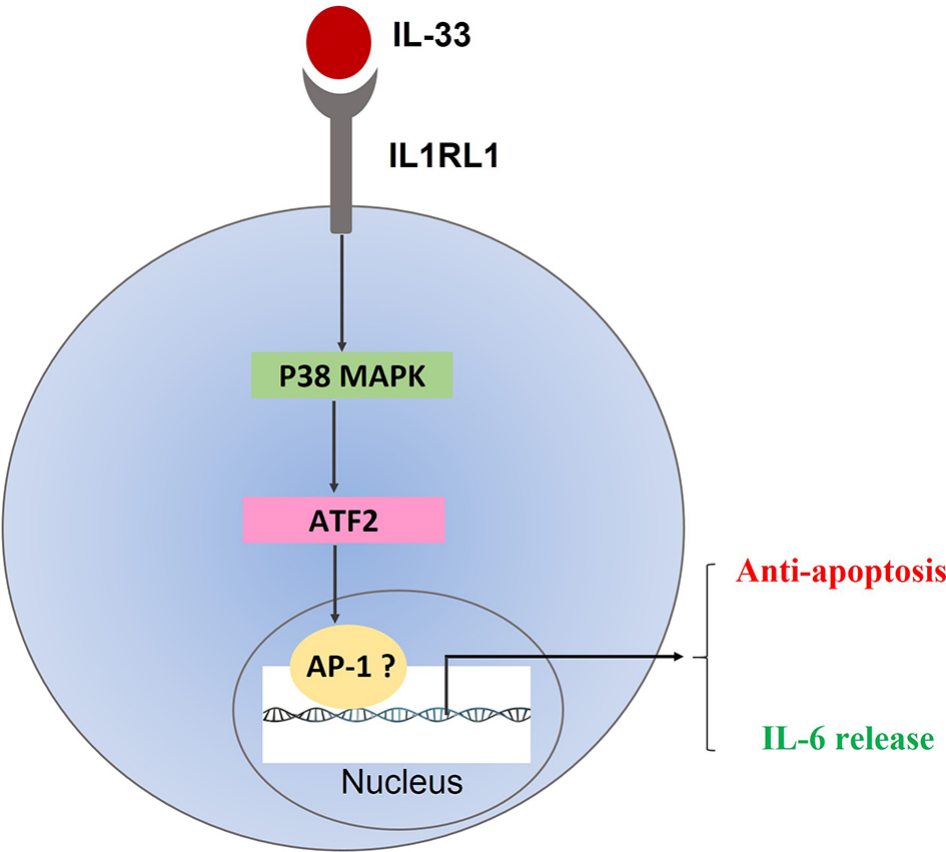
Proposed mechanism of IL33/IL1RL1 axis in mediating anti-apoptotic activity in primary human AML samples.

In conclusion, our study shows that the expressions of both IL-33 and its receptor IL1RL1 are elevated in primary pediatric AML samples. Furthermore, the data support a role for the p38 MAPK pathway in IL-33-mediated anti-apoptosis as well as IL-33-induced cytokine release. On the basis of these observations, we propose that IL-33/IL1RL1 axis is an important regulator for AML maintenance by activating p38 MAPK/ATF2 pathway, which could further promotes the transcriptional activities of AP-1 and leads to decreased apoptosis and increased IL-6 expression (**Figure 6**). Collectively, our work indicates that targeting the IL-33/p38 MAPK/IL-6 axis has the potential to be an effective treatment for pediatric AML patients.

## Data Availability Statement

The original contributions presented in the study are included in the article/**Supplementary Material**, further inquiries can be directed to the corresponding authors.

## Ethics Statement

The studies involving human participants were reviewed and approved by Guangzhou women and children’s medical center. Written informed consent to participate in this study was provided by the participants’ legal guardian/next of kin. 

## Author Contributions

YW, HS, MY, XG, and QG designed the study. YW, HS, QL, and JT performed the experiments and analyzed the data. MY and LZ collected the clinical data. YW, HS, MY, XG, and QG wrote the manuscript. All authors contributed to the article and approved the submitted version.

## Funding

This work was supported by grants from the Department of Education of Guangdong Province (2019KQNCX114) to YW and the Guangzhou Municipal Science and Technology Project (201904010067) and Natural Science Foundation of Guangdong Province (2018A030313560, 2020A1515010009) to QG.

## Conflict of Interest

The authors declare that the research was conducted in the absence of any commercial or financial relationships that could be construed as a potential conflict of interest.
