# Supplementary material for: Interleukin-33 Promotes Cell Survival via p38 MAPK-Mediated Interleukin-6 Gene Expression and Release in Pediatric AML
Source: Front Immunol. 2020 Nov 26;11:595053. doi: 10.3389/fimmu.2020.595053 (PMC7726021; doi:10.3389/fimmu.2020.595053)
Supplement: Supplementary file 1 [file Presentation_1.pptx]

## Slide 1
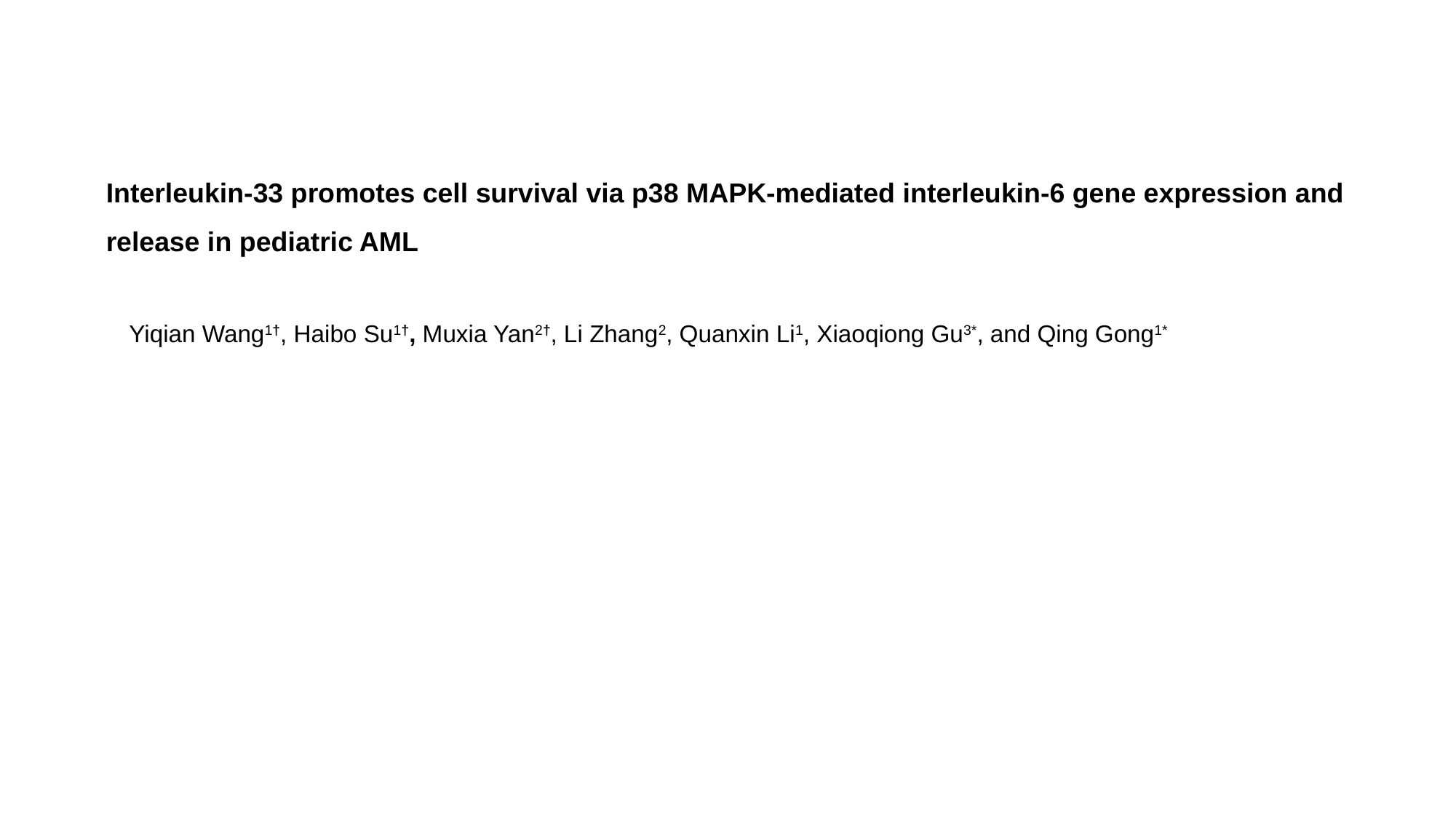

Interleukin-33 promotes cell survival via p38 MAPK-mediated interleukin-6 gene expression and release in pediatric AML
Yiqian Wang1†, Haibo Su1†, Muxia Yan2†, Li Zhang2, Quanxin Li1, Xiaoqiong Gu3*, and Qing Gong1*

## Slide 2
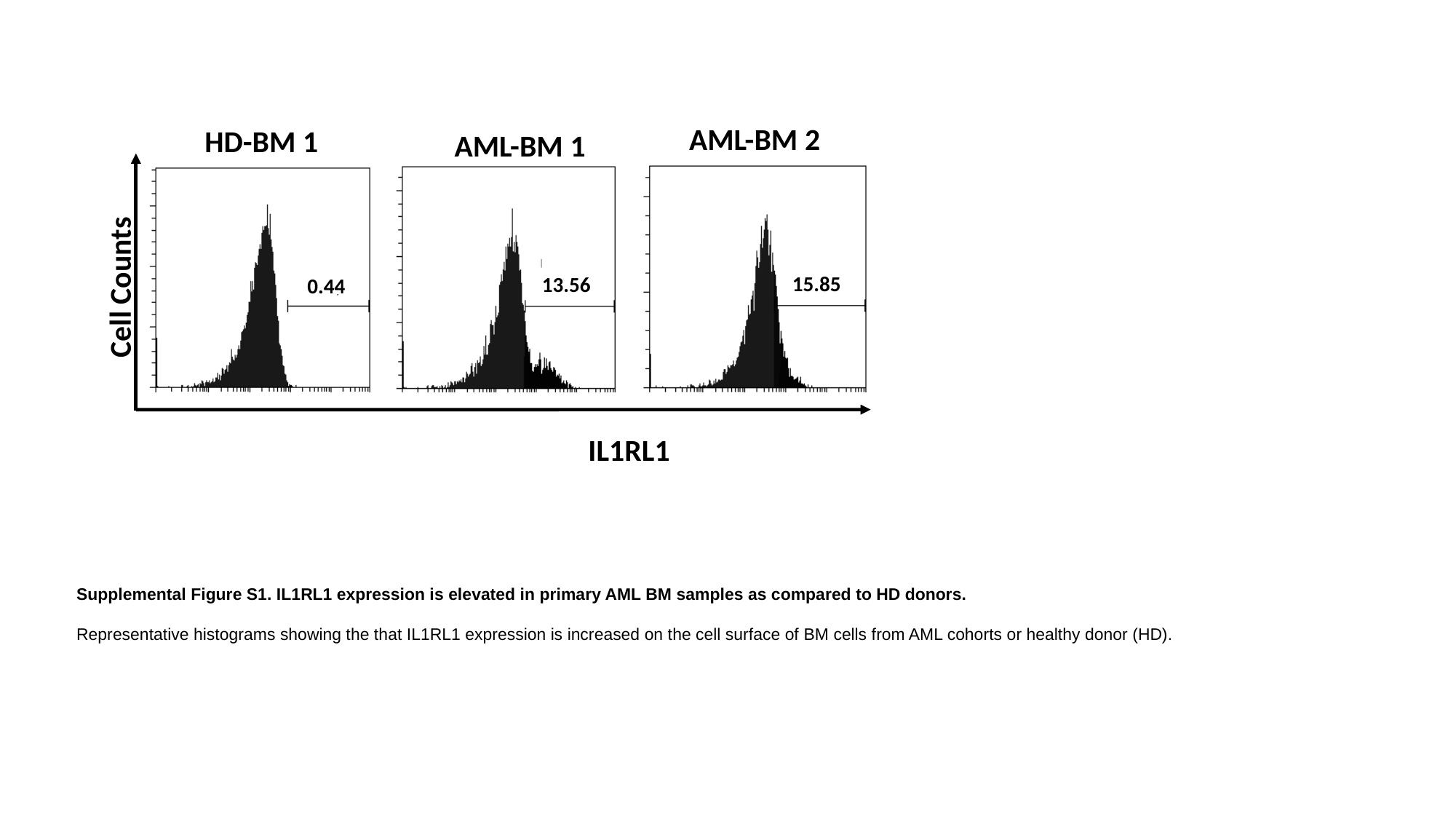

Cell Counts
IL1RL1
13.56
0.44
15.85
AML-BM 2
HD-BM 1
AML-BM 1
Supplemental Figure S1. IL1RL1 expression is elevated in primary AML BM samples as compared to HD donors.
Representative histograms showing the that IL1RL1 expression is increased on the cell surface of BM cells from AML cohorts or healthy donor (HD).

## Slide 3
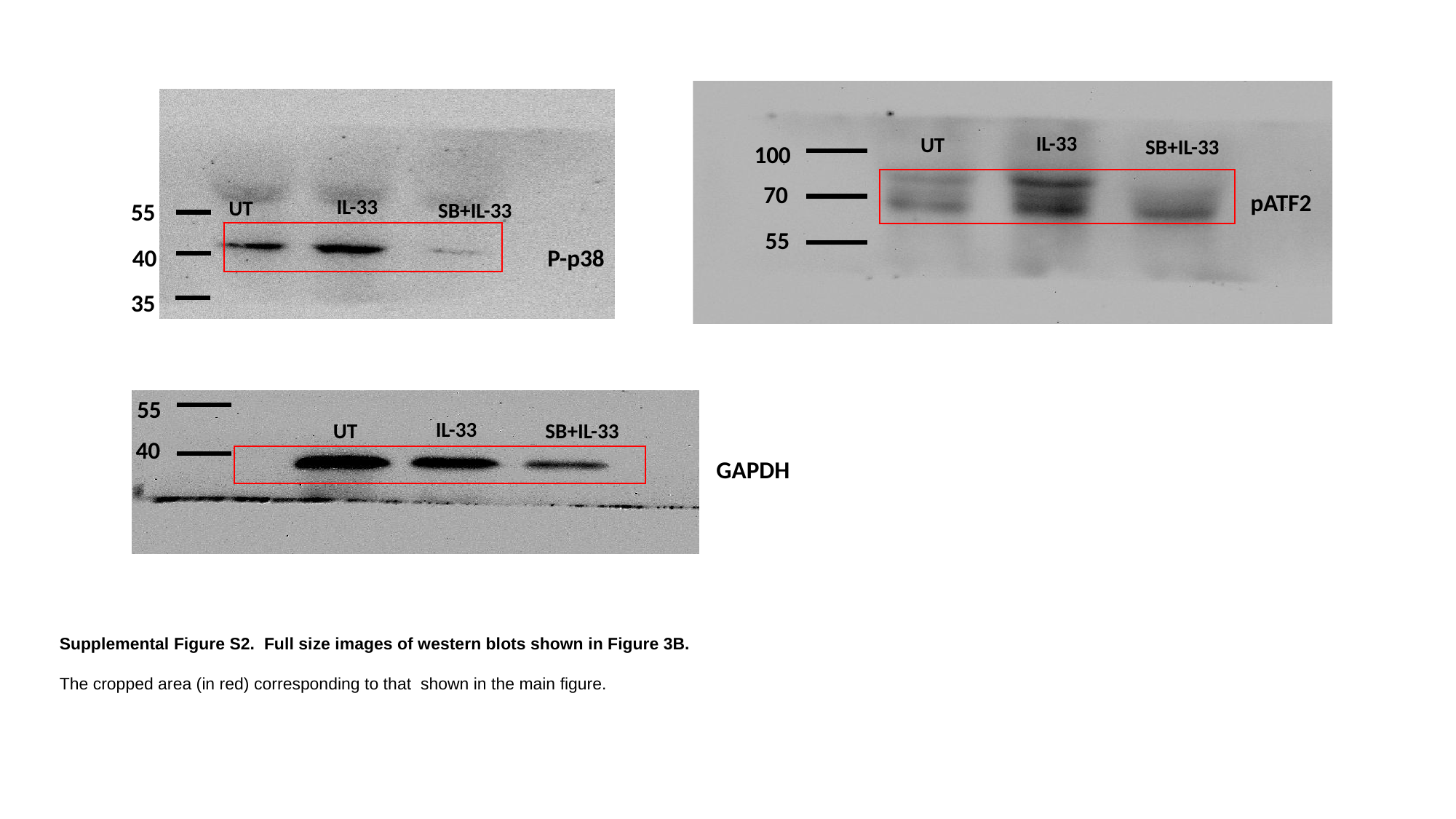

IL-33
UT
SB+IL-33
100
70
pATF2
55
IL-33
UT
SB+IL-33
55
40
35
P-p38
55
40
IL-33
UT
SB+IL-33
GAPDH
Supplemental Figure S2. Full size images of western blots shown in Figure 3B.
The cropped area (in red) corresponding to that shown in the main figure.

## Slide 4
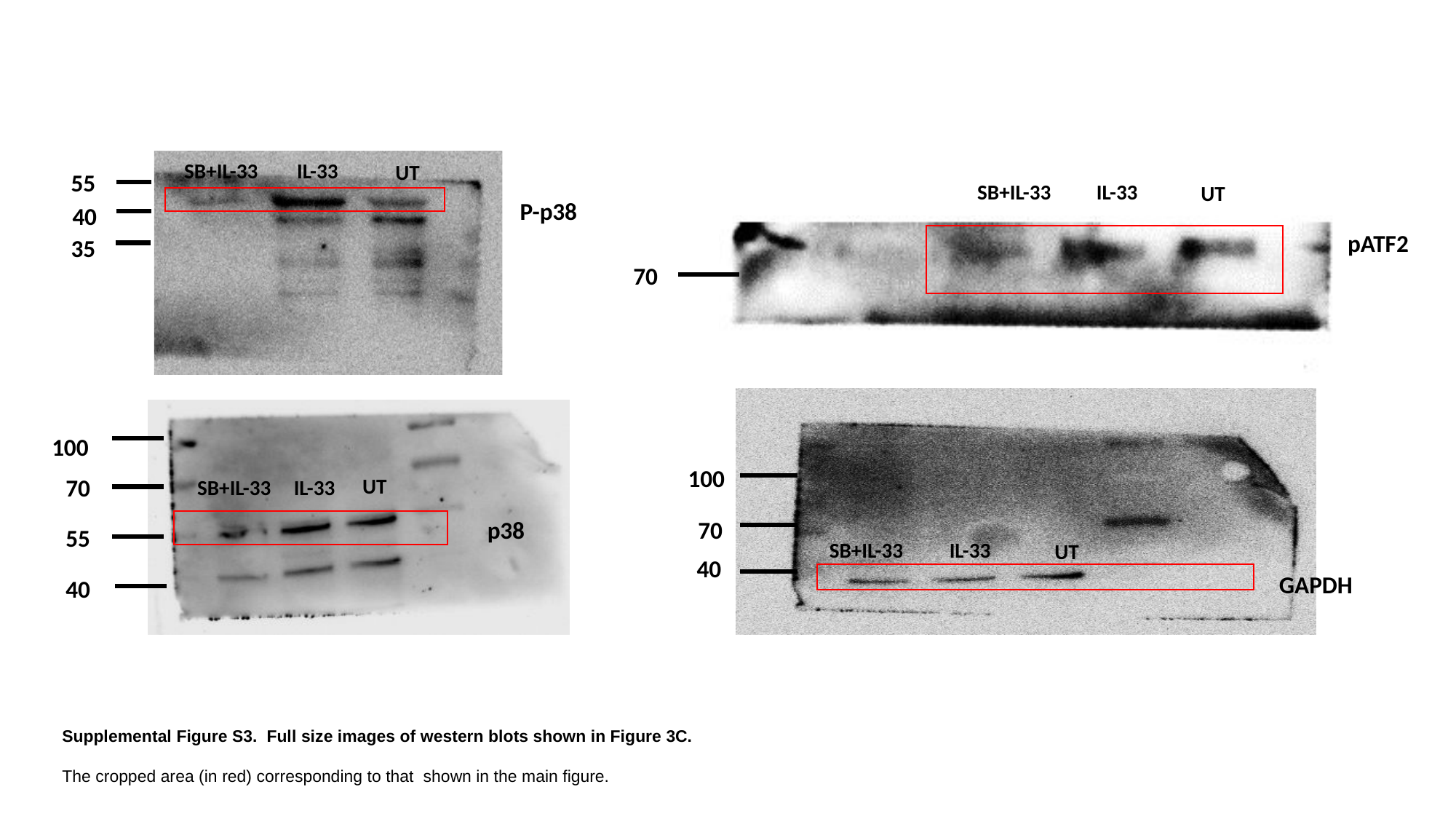

IL-33
SB+IL-33
UT
55
40
35
P-p38
IL-33
SB+IL-33
UT
pATF2
70
100
70
40
IL-33
SB+IL-33
UT
GAPDH
100
70
55
40
p38
UT
IL-33
SB+IL-33
Supplemental Figure S3. Full size images of western blots shown in Figure 3C.
The cropped area (in red) corresponding to that shown in the main figure.

## Slide 5
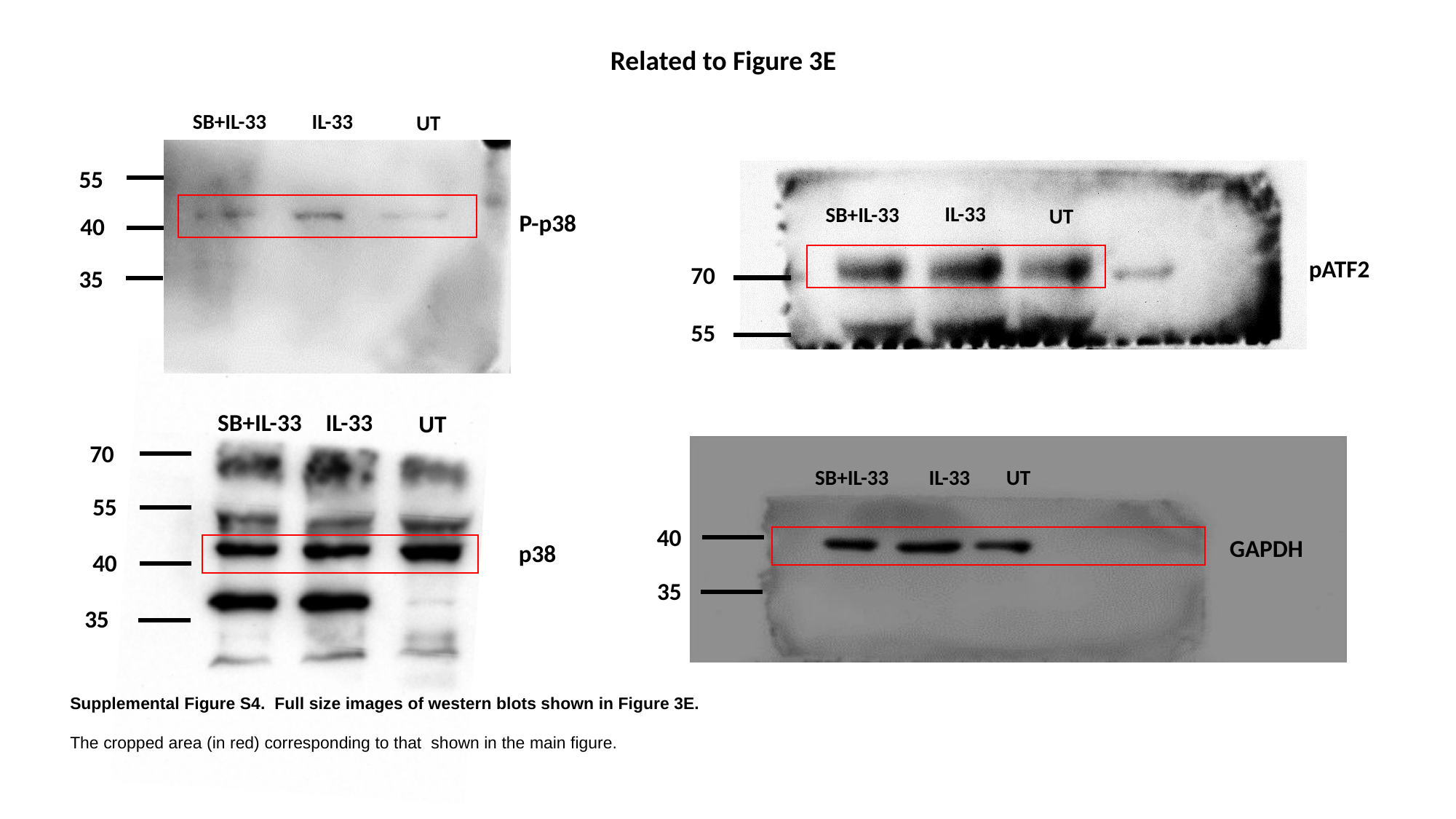

Related to Figure 3E
IL-33
SB+IL-33
UT
55
40
35
P-p38
IL-33
SB+IL-33
UT
pATF2
70
55
70
55
40
35
p38
IL-33
SB+IL-33
UT
40
35
GAPDH
SB+IL-33
IL-33
UT
Supplemental Figure S4. Full size images of western blots shown in Figure 3E.
The cropped area (in red) corresponding to that shown in the main figure.
